# AID Overlapping and Polη Hotspots Are Key Features of Evolutionary Variation Within the Human Antibody Heavy Chain (IGHV) Genes

**DOI:** 10.3389/fimmu.2020.00788

**Published:** 2020-04-30

**Authors:** Catherine Tang, Davide Bagnara, Nicholas Chiorazzi, Matthew D. Scharff, Thomas MacCarthy

**Affiliations:** ^1^Department of Applied Mathematics and Statistics, Stony Brook University, Stony Brook, NY, United States; ^2^Karches Center for Oncology Research, The Feinstein Institute for Medical Research, Northwell Health, Manhasset, NY, United States; ^3^Department of Experimental Medicine, University of Genoa, Genoa, Italy; ^4^Department of Cell Biology, Albert Einstein College of Medicine, Bronx, NY, United States

**Keywords:** somatic hypermutation (SHM), activation induced deaminase (AID), immunoglobulin heavy chain, computational immunology, B cell receptor (BCR), dimensionality reduction, unsupervised learning

## Abstract

Somatic hypermutation (SHM) of the immunoglobulin variable (IgV) loci is a key process in antibody affinity maturation. The enzyme activation-induced deaminase (AID), initiates SHM by creating C → U mismatches on single-stranded DNA (ssDNA). AID has preferential hotspot motif targets in the context of WRC/GYW (W = A/T, R = A/G, Y = C/T) and particularly at WGCW overlapping hotspots where hotspots appear opposite each other on both strands. Subsequent recruitment of the low-fidelity DNA repair enzyme, Polymerase eta (Polη), during mismatch repair, creates additional mutations at WA/TW sites. Although there are more than 50 functional immunoglobulin heavy chain variable (IGHV) segments in humans, the fundamental differences between these genes and their ability to respond to all possible foreign antigens is still poorly understood. To better understand this, we generated profiles of WGCW hotspots in each of the human IGHV genes and found the expected high frequency in complementarity determining regions (CDRs) that encode the antigen binding sites but also an unexpectedly high frequency of WGCW in certain framework (FW) sub-regions. Principal Components Analysis (PCA) of these overlapping AID hotspot profiles revealed that one major difference between IGHV families is the presence or absence of WGCW in a sub-region of FW3 sometimes referred to as “CDR4.” Further differences between members of each family (e.g., IGHV1) are primarily determined by their WGCW densities in CDR1. We previously suggested that the co-localization of AID overlapping and Polη hotspots was associated with high mutability of certain IGHV sub-regions, such as the CDRs. To evaluate the importance of this feature, we extended the WGCW profiles, combining them with local densities of Polη (WA) hotspots, thus describing the co-localization of both types of hotspots across all IGHV genes. We also verified that co-localization is associated with higher mutability. PCA of the co-localization profiles showed CDR1 and CDR2 as being the main contributors to variance among IGHV genes, consistent with the importance of these sub-regions in antigen binding. Our results suggest that AID overlapping (WGCW) hotspots alone or in conjunction with Polη (WA/TW) hotspots are key features of evolutionary variation between IGHV genes.

## Introduction

The affinity maturation of antibodies is a key part of the immune response against infectious diseases and vaccines. In the dark zone of the Germinal Center (GC), B cells undergo somatic hypermutation (SHM) of the immunoglobulin (Ig) variable (V) regions, accumulating mostly point mutations. As they move to the GC light zone, these B cells are also subject to selection with a preference for those B cells that gained favorable mutations leading to higher affinity binding B cell receptors (BCRs) (1–3). Expression of the mutagenic enzyme activation-induced deaminase (AID) in activated B cells is necessary to initiate SHM (4) by deaminating cytosine (C) to uracil (U) in single-stranded DNA (ssDNA) (5). Subsequent downstream error-prone DNA repair pathways—non-canonical base-excision repair (ncBER) and mismatch repair (ncMMR)—may be activated following the initial deamination event (6). As a consequence, mutations arise at the site of the original U:G mismatch due either to bypass during replication or BER involving Uracil DNA glycosylase (UNG), and at surrounding adenine (A) and thymine (T) bases due to MMR mediated by the low-fidelity polymerase Polη (7–9).

Previous studies of SHM data have shown that mutations are strongly biased toward particular IgV sites and sub-regions in several ways. Most clearly, mutations are biased toward the CDRs that correspond to the antigen binding sub-regions of the antibody protein, rather than the structural FW regions (10–12). Consistent with this, previous phylogenetic analyses of Ig heavy chain V (IGHV) genes in both human and mouse suggested there was stronger diversifying selection at the evolutionary level within CDRs compared to FWs (13, 14). Early work had also found that codons within the CDRs are more likely to lead to amino acid changes than codons for the same amino acid in the FW regions (15, 16). More recent studies have extended these results for all codons using approaches that take into account relative mutability (17, 18). As more antibody sequence data have become available, several studies have considered the importance of selection during SHM and have shown that intrinsic mutational bias is surprisingly strong relative to selection effects. For example, a comparison of non-productive sequences, which reflect the underlying mutation process in the absence of selection, with productive sequences from the human IGHV3-23 gene, found that the mutation profiles were highly similar (19), highlighting the importance of the intrinsic mutational bias.

At the micro-sequence level, it has been known for a long time that there exist sequence motifs, or “hotspots,” that are preferentially targeted during SHM (20). When AID was discovered and characterized biochemically, its preferential targeting to ssDNA with the motif WRC/GYW (W = A/T, R = A/G, Y = C/T) was confirmed, where the underlined base indicates the mutated site on the upper and lower strand respectively (21–23). Subsequent studies by ourselves (24) and others (25–27) confirmed the importance of overlapping AID hotspots (defined by the motif WGCW) in attracting mutations, particularly during the earliest rounds of SHM. In addition, mutations found in A:T bases stemming from WA/TW motifs are hallmark footprints left by Polη during non-canonical MMR (ncMMR) (9, 28). We further noted, at least for the IGHV3-23^*^01 gene, that the most highly mutated sub-regions, such as the CDRs, appeared to have both WGCW (e.g., AGCT) hotspots and a high density of Polη hotspots, and that mutations in the overlapping AID hotspots were associated with increased mutation levels throughout the whole V region (9, 24, 26, 28), which raised the possibility that certain WGCW sites might behave as “activation” sites for attracting AID and enabling subsequent mutations to these sub-regions. Based on these possibilities, we hypothesized that the difference in distribution and abundance of AID overlapping and Polη hotspots in the germline sequence of the different human IGHV genes, and their relationship to the frequency and distribution of mutations, will provide new insights into how we generate antibody diversity. Note that we will refer to the preferred motifs for both AID and Polη as “hotspots” throughout, since this is how they are conventionally referred to, although this does not necessarily mean each such “hotspot” is highly mutated.

In this study, we began by analyzing the human IGHV genes based solely on their distribution of AID overlapping (WGCW) hotspots using a moving window approach. Interestingly, WGCW sites are found at high frequency not only in the CDRs, as one would expect, but also in two FW sub-regions. Using dimensionality reduction—in this case Principal Components Analysis (PCA) which uses statistical learning to transform a large set of possibly correlated variables into a new set of meaningful and uncorrelated variables—we found that the inter-family differences in IGHV genes are characterized by the differential distribution of AID overlapping hotspots in two FW sub-regions: one at the 5′ end of FW1; and the other in a sub-region of FW3 known informally as “CDR4” (29). At the same time, intra-family differences can be also described in terms of variation of WGCW hotspots in CDR1. This apparent difference in intra-family and inter-family variation is particularly interesting since it is unclear why most of the human variable region families have only 1–12 members while the IGHV3 family has 25 productive germline genes and several non-productive genes. Based on these findings, we sought to develop a strategy for characterizing and comparing all of the human IGHV genes based on a simple profile that uses the co-localization of both AID overlapping and Polη WA/TW hotspots to account for possible interactions between AID and MMR. PCA of these profiles revealed that variation within the IGHV genes can also be characterized in terms of the germline distribution of co-localized AID overlapping and Polη hotspots observed mostly within CDR1 and CDR2, and to an extent FW3. With the inclusion of non-functional IGHV genes (e.g., pseudogenes), we observed markedly reduced co-localized hotspots in CDR1 and CDR2 while the numbers in FW3 remain the same compared to functional IGHV genes.

## Materials and Methods

### Human Datasets

Data from the memory, marginal zone, and plasma cell subsets (B10-B14, B16-21, HD001-10) from 21 healthy individuals from NCBI SRA BioProjects 381394 and 591804 were used in this study.

### Data Preprocessing

Datasets were processed using pRESTO, Change-O, and TIgGER toolkits, part of the Immcantation framework (https://immcantation.readthedocs.io/en/version-2.6.0/). As previously described in Vergani et al. (30), barcoded (labeled with Unique Molecular Identifier, UMI) Illumina MiSeq sequences were assembled using pRESTO, with low quality reads (*Q* < 20) and reads observed only once (CONSCOUNT <2) being removed. Resulting FASTA files were then submitted to IMGT/High-VQuest to identify IGHV gene assignments and CDR3 boundaries. In order to avoid possible effects of selection, only sequences identified as non-productive due to frameshifts or stop codons in CDR3 by IMGT were used, since such rearranged V regions were non-productive from the time of VDJ rearrangement (31). The Change-O package was then used to determine clonal groups based on the CDR3 sub-region, separately for each individual dataset (each corresponding to a separate human individual). To avoid issues arising from clonality, we randomly selected one sequence per clone for the analysis. Only V segments, excluding the CDR3 and J, were used in the following analysis. We also used the TIgGER package to identify possible novel (non-IMGT) alleles. Any sequences assigned to novel alleles were removed in order to avoid the associated polymorphisms generating false-positive “mutations” (32). All subsequent analysis was performed using custom R scripts.

### Germline Sequence Data

The human germline IGHV genes used in the analysis were downloaded from the international ImMunoGeneTics information system (IMGT) website (www.imgt.org). We distinguished CDR and FW boundaries according to the unique IMGT numbering scheme. Gapped germline sequences were truncated to 294 nt to avoid possible nucleotide addition at the junction of FW3 and CDR3 in our dataset.

### Generating Hotspot Profiles for All IGHV Genes

The starting point for our analysis is the distributions of AID WGCW and Polη WA/TW hotspots (Figures S1, S2 for the IGHV3 family, and other the 6 IGHV families, respectively). To generate the hotspot profiles for each IGHV germline gene, as described in the main text, we used a moving window of size 15 nt both upstream and downstream of each nucleotide position (for a total window size of 31), counting the number of hotspots of interest, and then dividing by the total window size. In other words, each sequence is represented as a hotspot distribution profile where each value measures the hotspot density in the neighborhood around each position in the sequence. To ensure that the distribution profiles were of equal length for subsequent analyses (see below), we used the standard gapped alignments from IMGT and linear interpolation, a curve-fitting method, to adjust for differences in IGHV sequence lengths using the R function *approx*. Lastly, allelic variants were ignored and only ^*^01 alleles were used except where indicated otherwise. For Principal Components Analysis (PCA), the hotspot distribution profiles were used as input, which was performed using the R function *prcomp*.

### Correction for Hotspot Composition in the WGCW/WA Profiles

As expected due to their lower motif complexity, WA hotspots are more abundant, making the scale of their profile generally higher in absolute terms. Thus, before calculating the product of the two profiles, we rescaled the WA/TW profiles by 1/12, which corresponds to the difference in expected frequency given a random and equiprobable nucleotide background (frequency of WA or TW: 3/(4^2^) = 3/16; frequency of WGCW: 4/(4^4^) = 1/64). As before, we used linear interpolation to ensure all profiles have the same sequence length.

### Classifying WGCW/WA Sub-regions

We used thresholding to discretize (convert to TRUE/FALSE values) the profiles describing co-localization of AID WGCW overlapping and Polη WA/TW hotspots (see Figure S3). In other words, we defined a cutoff value that classified each site of every distribution profile as either falling within (TRUE), or outside (FALSE) the WGCW/WA co-localization sub-region. When deciding on a threshold value we observed that if the threshold was set too low then, for certain V genes, both CDRs would be entirely above the threshold, leaving no below-threshold sub-region to compare with, in which case the V gene would have to be removed from the analysis. On the other hand, if the threshold was set too high, then for some V genes there would be no sites recognized as belonging to the WGCW/WA sub-region, particularly in FW regions, again leading to removal of these genes. We identified an optimal range of threshold values for which there was the least number of V genes removed due to either of these effects, and then defined the midpoint of this range as the threshold value for further analysis. With the sites for each V gene thus discretized, we counted mutations falling into the WGCW/WA co-localization sub-region and those outside separately. If there were not enough sequences from the ^*^01 allele, then alternative alleles were used if these had enough data—in practice the only alternative alleles used were IGHV1-2^*^02, IGHV3-49^*^03, IGHV4-4^*^02, and IGHV5-10-1^*^03 for CDRs; and IGHV1-45^*^02, IGHV3-49^*^03, and IGHV4-4^*^02 for FW regions. To test for significance of mutations within WGCW/WA sub-regions, we implemented a one-sided binomial test using the R function *binom.test* using the proportion of sites within the WGCW/WA co-localization sub-region as the parameter for expected *p*. The resulting FDR-corrected binomial test *P*-values (Benjamini-Hochberg method) were calculated using the R function *p.adjust*.

## Results

### Overlapping Hotspot Densities Vary Greatly Between Multiple Sub-regions of the V Region

**Figure 1:**
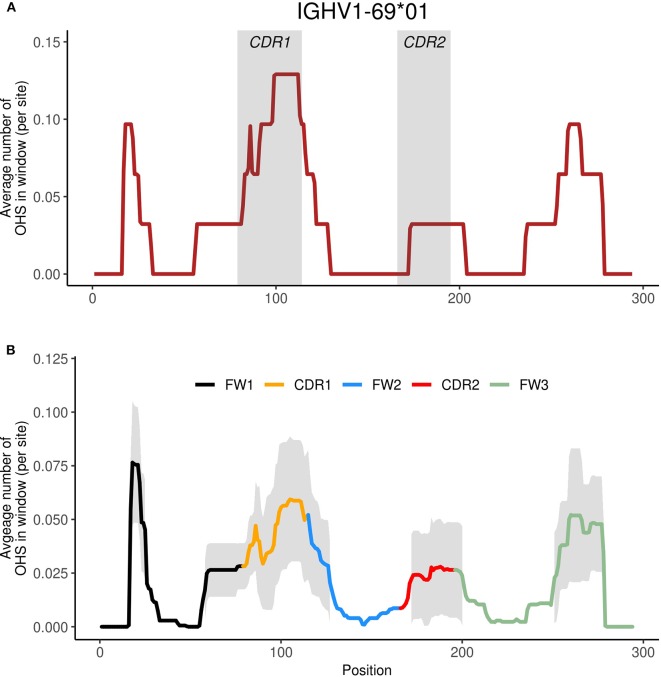
Identifying WGCW hotspot regions. **(A)** We show the moving window profile for WGCW overlapping AID hotspots for IGHV1-69. The shaded areas mark CDR1 and CDR2. **(B)** Site-by-site calculation of the average number of WGCW hotspots found in a window of size 31 (+/– 15 nt around each site). The bold line indicates the average across the 56 human IGHV genes and is colored according to sub-region. The shaded region represents +/– 1 standard deviation at each site.

Given the importance of overlapping hotspots as prime sites of AID targeting (21–23, 25), as well as their influence on enhanced mutability throughout the V region in IGHV3-23^*^01 as we had observed previously (24), we now examined all functional human IGHV genes to identify similarities or differences in terms of overlapping AID hotspot distributions. We constructed profiles for each IGHV gene using a sliding window of +/– 15 nt around each position to describe the surrounding frequency of overlapping AID hotspots (see Materials and Methods). As an example, Figure 1A shows the WGCW profile for IGHV1-69^*^01, one of the most commonly used human IGHV genes, and which also contains a high number of WGCW sites. The window size of +/– 15 nt was chosen to be comparable to the size of a typical CDR 1 or 2 sub-region (maximum widths using IMGT scheme, CDR1: 36 nt, CDR2: 30 nt), although varying this parameter does not qualitatively change the results as will be described below (Figure S4). Figure 1B shows the mean and one standard deviation of these profiles for the set of 56 functional human IGHV genes in IMGT. The highest average level of overlapping AID hotspots was located in a small sub-region of highly conserved WGCW sites at the 5′ end of FW1. This is a highly conserved cluster of overlapping AID hotspots in all families except IGHV2 (Figures S1, S2) whose presence is rarely recognized and function is not known. The next two largest peaks, which are shared by all of the IGHV genes and are roughly of equal magnitude, are found in CDR1 and, somewhat surprisingly, in FW3, as opposed to CDR2, which has a mean density roughly half that of CDR1 (Figure 1B). Interestingly, CDR1 contains an additional lower peak, driven by a single AID overlapping hotspot just 5′ to CDR1 in almost all of the IGHV3, IGHV1, and IGHV2 family members (Figure 1B; Figures S1, S2). The relatively high density of overlapping hotspots in FW1 and FW3 is, in a way, unexpected given that FW regions generally have lower mutation frequencies than the CDRs. We further noted that the peaks were more-or-less evenly spaced at intervals of ~85 nt. Also, the variance was high in all four sub-regions, suggesting that over evolutionary time, substantial differences have arisen between the IGHV genes in terms of the frequency of AID overlapping hotspots within each sub-region.

### FW1 and FW3 Display a Greater Contribution to Overlapping Hotspot Variation Than the Other Sub-regions

**Figure 2:**
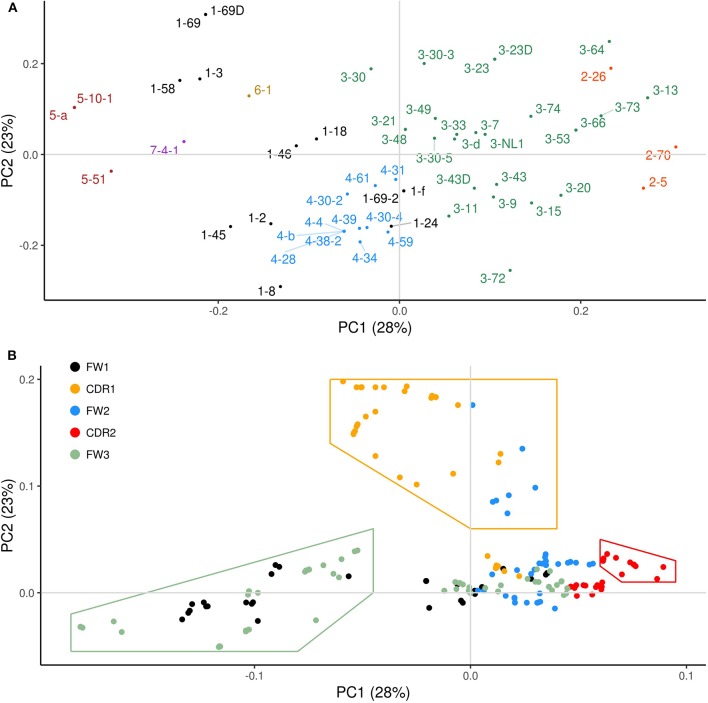
Principal components analysis (PCA) of functional human IGHV genes. **(A)** PCA transformation of the WGCW hotspot distribution profiles for 56 functional human IGHV genes analyzed, known as PCA scores, with respect to the first two principal components (PC1 and PC2). The amount of variance from the WGCW hotspot distribution profiles captured by each PC is shown in parentheses. Each gene is colored according to its corresponding IGHV family. Gene labels located far from their corresponding dot are attached by a fine line to overcome the problems of overlapping and nearby numbers. **(B)** PCA loadings plot where each dot represents a site and its relative contribution to each of the first two PCs. Distance from the origin (where PC1 and PC2 intersect) signifies the magnitude of each site's loadings contribution. Colors indicate the sub-region of each site. Dots enclosed by colored lines indicate high-contributing sites for each category (CDR1, CDR2, FW3).

In order to identify common patterns of overlapping AID hotspots among the IGHV genes and to better describe associations between the sub-regions, we performed Principal Components Analysis (PCA) using the overlapping hotspot distribution profiles for each gene (see Materials and Methods). The newly transformed variables resulting from PCA, called principal components (PCs), are calculated such that the most variation (maximum variance) of the WGCW distribution profiles is captured by the first PC, the second PC explaining the next most variation from the remaining variance, and so on. Another useful feature of PCA is that all PCs are uncorrelated with one another. The transformed data are commonly shown projected onto a plot using PC1 (x-axis) and PC2 (y-axis), crossing at the origin (i.e., where the axes intersect), where data points that have similar features are expected to cluster together. Figure 2A, known as the PCA scores, shows such a plot. Here, each of the IGHV genes, colored by gene family, are projected onto the first two PCs, which reflect the two major independent variances in the distributions of the WGCW overlapping AID hotspots. These projections illustrate how individual IGHV genes are distributed spatially with respect to PC1 and PC2.

Figure 2B shows the corresponding PCA loadings, which describe the contribution of each site on the sequence (represented by color-coded dots) to the overall variation. The magnitude of the PCA loadings, either positive or negative, quantifies the strength of their contribution to the variance, so loading values far from the origin represent larger contributions whereas those close to the origin are smaller. In the loadings plot in Figure 2B, each dot represents a site and is color-coded according to the sub-region (CDR1-2, FW1-3). If we consider sites that are far from the origin, we observe a clear separation between the CDR1 sites (orange) on the one hand, and FW1 (black) and FW3 (green) together on the other. CDR2 sites (red) also appeared along PC1 in the opposite direction to the FW1 and FW3 sites, albeit to a lesser degree. Because we observed that CDR1 loadings largely track with PC2 and that FW1 and FW3 loadings track with PC1 (as do, to a lesser extent, those of CDR2), it suggests that CDR1 variation is independent (uncorrelated) with both variation in FW1 and FW3, and CDR2.

To explore these independences of variation further, we selected sets of sites along PC1 or PC2 with loadings that are furthest from the origin and contribute most to the variation as indicated by the areas enclosed by the lines in Figure 2B, which are colored according to the most abundant sub-region (CDR1, CDR2, or FW3) within each set. Note that some FW2 sites (blue) that are adjacent to the 3′ end of CDR1 were included as part of the CDR1 set (Figure 2B; Figures S1, S2). We then calculated, for each gene separately, the mean overlapping hotspot frequency for each set of sites (here FW1 and FW3 sites were at first considered separately), giving four mean values for each of the 56 genes. Pairwise correlations between the four sets (CDR1, CDR2, FW1, FW3) were then calculated (Figure S5). As expected, FW1 and FW3 were positively correlated (Figure S5A; Pearson's ρ = 0.76, *P* = 1.34 × 10^−11^), so we combined these sites into one set (FW1/3) thereafter. On the other hand, we found that the FW1/3 set was negatively correlated with the CDR2 set (Figure S5B; Pearson's ρ = −0.52, *P* = 3.92 × 10^−5^). There was also no significant correlation between the CDR1 set and the CDR2 set (Figure S5C; Pearson's ρ = 0.08, *P* = 0.568), as well as between the CDR1 set and the FW1/3 set (Figure S5D; Pearson's ρ = 0.08, *P* = 0.535). Because PC1 explains more variation than PC2, and because sites in FW1/3 make the largest PC1 contributions, our results suggest that the presence or absence of overlapping hotspots in FW1 and FW3 explain major differences in IGHV genes. CDR1 is also a major contributor to variation, with patterns of overlapping hotspots that are independent of those of FW1/3.

### IGHV Families Group by Their Densities of Overlapping Hotspots in FW1 and FW3

The PCA loadings (Figure 2B) are useful in explaining how the IGHV genes have clustered in the PCA scores plot (Figure 2A). Along PC1 (the PC that explains the greatest amount of variance), IGHV genes largely group by family, as one might expect, with the exception of the IGHV1 family, for which the range includes the IGHV4 family cluster. As suggested by the pattern of loadings along PC1, these inter-family differences emerge due to the presence or absence of overlapping hotspots within FW1 and FW3 since IGHV genes that yield high negative contributions to PC1 will be those containing overlapping hotspots in FW1 and FW3 that are associated with negative loadings (Figure 2B). It is important to note that the sign of the loadings are, in a sense, arbitrary since changing their signs—from positive to negative, and vice versa—would not qualitatively change the results (i.e., the positional arrangement of IGHV genes would remain the same on the plot); rather it is the magnitude of the loading contribution that is more telling because it determines where in the scores plot (Figure 2A) the IGHV genes are positioned. To demonstrate this effect, IGHV1-69, for instance, contains three WGCW hotspots in CDR1 as well as another one just 3′ of CDR1; it also contains several overlapping hotspots in both FW1 and FW3 (Figure S2). Because those sites found further along negative PC1 (to the left of the origin) are associated with FW1 and FW3, and because CDR1 sites are generally found along positive PC2 (upward from the origin) (Figure 2B), any gene containing hotspots in both sub-regions, such as IGHV1-69, will be forced toward the upper-left quadrant of the scores plot (Figure 2A).

In addition, the relationships between the PCA scores and loadings can also be extended to entire gene families. Most obviously, the IGHV5 family genes, at the left side of PC1 (Figure 2A), have four overlapping hotspots located at the 5′ end of FW1 and three or more additional overlapping hotspots around positions 250–280 of FW3 (Figure S2). At the other extreme of PC1 (right side of Figure 2A), the IGHV2 family is exceptional in that it does not have any overlapping hotspots within the first 25 nt at the 5′ end of FW1 or in the hypervariable area of FW3 (Figure S2), which is again consistent with the corresponding loadings (green delimited area in Figure 2B). Furthermore, it is also important to know that loadings that are along the same PC but located in opposite directions from the origin, signify that they contribute to variation in opposing ways. Since we found sites in FW1/3 to be negatively correlated with sites in CDR2 (Figure S5B), we also expected to find differences between the IGHV2 and IGHV5 families with respect to overlapping hotspot(s) in CDR2. For example, there is a TGCA hotspot at the 3′ end of FW2, next to CDR2, that occurs only in the IGHV2 family (Figure S2).

The IGHV5 and IGHV2 gene families represent two extremes of a spectrum that explains a large amount of variation in overlapping hotspot densities between IGHV families, ranging from the greatest to the least number of overlapping hotspots contained in FW1 and FW3. The remaining IGHV families fall between these two extremes. For example, some members of the IGHV1 family, such as IGHV1-58, 1-69, and 1-69D, as well as the lone IGHV7 gene, IGHV7-4-1, are very similar to IGHV5 genes in that they contain three overlapping hotspots in FW1 as well as three more hotspots in FW3 (Figure S2). Of note, this particular sub-region of FW3 includes one of two previously reported non-CDR regions of hypervariability [nt positions 256–273 (29), and is also referred to as “CDR4”]. On the other hand, there are some IGHV3 family genes, such as IGHV3-53, 3-64, and 3-13, that are missing overlapping hotspots in the hypervariable sub-region of FW3 (Figure S1), and therefore, are found to be closer to the IGHV2 family.

### Differences Within IGHV Families Are Primarily Determined by Overlapping Hotspots in CDR1

Since varying densities of overlapping hotspots in FW1 and FW3 appear to determine differences between the IGHV families, we suspected that the remaining variation, which we had already found to be high in CDRs (Figure 1B), would be associated with intra-family differences. We began by analyzing the two largest IGHV families, IGHV3 and IGHV1, which account for approximately two-thirds (37/56) of the functional genes and appear to have more variable overlapping hotspot distributions in both CDRs as compared to the other gene families (Figures S1, S2).

We noted earlier that CDR1 is a region with a high density of overlapping AID hotspots and very high variance (Figure 1B). Given the loadings pattern (orange-delimited area in Figure 2B), we expected differences along PC2 to reflect the number of WGCW hotspots specifically in CDR1 and the 5′edge of FW2. Thus, genes such as IGHV3-30 and 3-23 that are in the positive PC2 direction (Figure 2A) contain at least one overlapping hotspot in CDR1, whereas genes such as IGHV3-72, that are in the negative PC2 direction, have no overlapping hotspots in CDR1 (Figure S1). The IGHV1 family is similar, with genes such as IGHV1-69 (top of Figure 2A) having many overlapping hotspots in CDR1 and others such as IGHV1-8 having none (bottom of Figure 2A; Figure S2). These examples are only a couple of the many other instances that highlight this sort of intra-family difference. IGHV genes belonging to the same family, combined with high variance primarily in CDR1 (as noted in Figure 1B), confirmed that CDR1 was the major source of intra-family variation.

### Clusters of Co-localized Polη Hotspots and AID Overlapping Hotspots Are Associated With Higher Mutation Frequencies

Our previous analysis of the IGHV3-23 gene (24) noted that the CDRs appeared to have a co-localization of overlapping AID hotspots, in this case AGCT, with clusters of WA/TW motifs. Mutations occurring in WA/TW hotspots are associated with the error-prone DNA repair enzyme, Polη, as part of the non-canonical mismatch repair (ncMMR) pathway which often acts downstream of AID-deamination at nearby C residues (3). As above where we considered only WGCW sites, we wanted to search for possible interactions between AID and Polη hotspots over a range that was at least as wide as CDR1 or CDR2 (again using +/– 15 nt) in all human IGHV genes (see below).

We first sought to identify potential sub-regions for heightened AID and Polη interactions by defining a formal, yet simple, measure to quantify this synergy across all IGHV genes. In a similar way to the AID overlapping hotspot distributions that we calculated previously (as shown in Figure 1A for IGHV 1-69, and repeated in Figure S3A), we calculated equivalent distribution profiles for Polη WA (TW on opposite strand) hotspots (Figure S3B) for all of the genes using the same method described above for characterizing WGCW motifs. We then defined a measure for the co-localization of both AID overlapping and Polη hotspots as the site-by-site product of the WGCW and WA/TW profiles (Figure S3C), correcting for the relatively greater abundance of WA/TW hotspots (see Materials and Methods). As a result, we have generated profiles for each IGHV gene that identify the sub-region(s) in each gene where there is a co-localization of both WGCW and WA/TW motifs.

Due to the presence of mutational hotspots, we obviously expected the sub-regions identified as containing both WGCW and WA/TW motifs to be functionally relevant and therefore more highly mutated *in vivo*. To verify this using mutation data, we began by categorizing each site either as belonging to a WGCW/WA sub-region or not using the co-localization profiles for each gene. We used a single threshold value to separate the two categories. The threshold was chosen so we could include as many V genes as possible (see Materials and Methods; Figure S3C). Next, we used a large dataset of high-quality IGHV repertoire data from non-productive, and therefore not antigen-selected sequences, to obtain mutation data for different human IGHV genes where each V region was represented by at least 100 sequences with multiple mutations (see Materials and Methods). We utilized the mutation data for the corresponding IGHV gene to count the mutations occurring both within and outside the WGCW/WA sub-regions.

**Figure 3:**
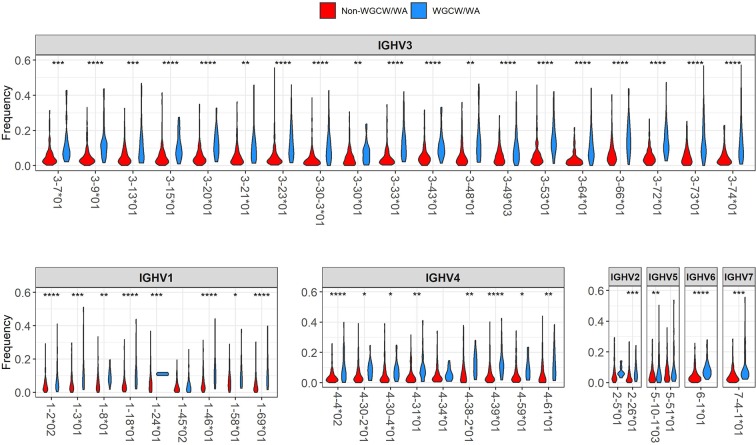
Distribution of mutation frequency between WGCW/WA sub-regions and non-WGCW/WA sub-regions. The distribution of the observed mutation frequencies is shown separately for WGCW/WA sub-regions (blue), and non-WGCW/WA sub-regions (red) for each individual IGHV gene. One-sided *t*-tests comparing the two distributions were performed for each gene. Significant *p*-values are indicated by asterisks above each plot (*p < 0.05; **p < 0.01; ***p < 0.001; ****p < 0.0001).

The violin plots in Figure 3 show, for each IGHV gene, the distribution of mutation frequencies for sites within the WGCW/WA sub-regions (blue distributions) and outside these sub-regions (red distributions). The mutation frequencies within the WGCW/WA sub-regions are significantly higher (using a *t*-test) for almost all IGHV genes (39/43) that we considered (Figure 3). As an alternative comparison, the mutation counts within each WGCW/WA sub-region were compared (using a binomial test, see Materials and Methods) to their expected value, defined as the relative proportion of sites found within the WGCW/WA sub-regions (Table 1). This comparison confirmed that WGCW/WA sites acquire more mutations than expected for all IGHV genes considered. Because CDRs would be expected to mutate more than FW regions and therefore be enriched for hotspots (CDRs contain on average 50.67% ± 20.67 WGCW/WA sites compared to 5.88% ± 4.22 for the FW regions), we also considered CDR and FW regions separately (Table S1 for CDRs; Table S2 for FWs). Note that for certain IGHV genes in this dataset, we found no observed mutations either within the WGCW/WA sub-region or, in other cases, outside the sub-region. In such cases, the genes were removed (independently for CDR and FW), leaving 40 and 41 IGHV genes for CDR and FW regions, respectively, to be used for this analysis. When considering only the CDR regions, we still found that the WGCW/WA sub-regions mutated at a significantly higher frequency than non-hotspot containing regions in 30 out of 40 (75%) IGHV genes (Table S1). Similarly, when we considered the FW regions, we found a significantly higher frequency in 29 of the 41 (71%) of IGHV genes (Table S2). Furthermore, we consider these results to be conservative since for some genes, the percentage of WGCW/WA sites is very high (e.g., 45/48 sites = 93.8% for IGHV3-23 in Table S1), leaving only a small number of sites to compare against, which in turn reduces statistical power giving the false impression that the WGCW/WA sub-regions were not more highly mutated in that gene.

**Table 1:** Binomial test results showing mutability of all WGCW/WA sites (CDRs + FWs).

**Allele**	**Num mutations within WGCW/WA sites**	**Num mutations outside WGCW/WA sites**	**Total num mutations**	**Percent mutations within WGCW/WA regions**	**Num WGCW/WA sites**	**IGHV gene length (ungapped)**	**Percent WGCW/WA sites**	***P*-value**	**Corrected *P*-value**
IGHV1-2*02	960	13,928	14,888	6.4	10	270	3.7	*P* < 10^−20^	*P* < 10^−20^
IGHV1-3*01	2,380	7,684	10,064	23.6	31	270	11.5	*P* < 10^−20^	*P* < 10^−20^
IGHV1-8*01	907	7,931	8,838	10.3	18	270	6.7	*P* < 10^−20^	*P* < 10^−20^
IGHV1-18*01	7,472	11,325	18,797	39.8	57	270	21.1	*P* < 10^−20^	*P* < 10^−20^
IGHV1-24*01	76	3,840	3,916	1.9	3	270	1.1	4.52 × 10^−6^	4.86 × 10^−6^
IGHV1-45*02	75	1,738	1,813	4.1	8	270	3.0	3.04 × 10^−3^	3.11 × 10^−3^
IGHV1-46*01	4,032	7,368	11,400	35.4	51	270	18.9	*P* < 10^−20^	*P* < 10^−20^
IGHV1-58*01	459	2,153	2,612	17.6	33	270	12.2	1.56 × 10^−15^	1.77 × 10^−15^
IGHV1-69*01	3,399	5,778	9,177	37.0	54	270	20.0	*P* < 10^−20^	*P* < 10^−20^
IGHV2-5*01	130	3,706	3,836	3.4	7	273	2.6	1.14 × 10^−3^	1.19 × 10^−3^
IGHV2-26*01	956	2,896	3,852	24.8	43	273	15.8	*P* < 10^−20^	*P* < 10^−20^
IGHV3-7*01	4,731	12,711	17,442	27.1	40	270	14.8	*P* < 10^−20^	*P* < 10^−20^
IGHV3-9*01	5,192	16,114	21,306	24.4	30	270	11.1	*P* < 10^−20^	*P* < 10^−20^
IGHV3-13*01	4,349	9,487	13,836	31.4	48	267	18.0	*P* < 10^−20^	*P* < 10^−20^
IGHV3-15*01	2,546	6,362	8,908	28.6	45	276	16.3	*P* < 10^−20^	*P* < 10^−20^
IGHV3-20*01	764	2,441	3,205	23.8	32	270	11.9	*P* < 10^−20^	*P* < 10^−20^
IGHV3-21*01	3,446	13,941	17,387	19.8	28	270	10.4	*P* < 10^−20^	*P* < 10^−20^
IGHV3-23*01	17,854	31,432	49,286	36.2	54	270	20.0	*P* < 10^−20^	*P* < 10^−20^
IGHV3-30-3*01	3,907	4,442	8,349	46.8	68	270	25.2	*P* < 10^−20^	*P* < 10^−20^
IGHV3-30*01	751	2,813	3,564	21.1	40	270	14.8	*P* < 10^−20^	*P* < 10^−20^
IGHV3-33*01	6,621	12,254	18,875	35.1	45	270	16.7	*P* < 10^−20^	*P* < 10^−20^
IGHV3-43*01	908	1,869	2,777	32.7	52	270	19.3	*P* < 10^−20^	*P* < 10^−20^
IGHV3-48*01	1,052	4,097	5,149	20.4	28	270	10.4	*P* < 10^−20^	*P* < 10^−20^
IGHV3-49*03	1,432	1,737	3,169	45.2	82	276	29.7	*P* < 10^−20^	*P* < 10^−20^
IGHV3-53*01	2,170	5,770	7,940	27.3	35	267	13.1	*P* < 10^−20^	*P* < 10^−20^
IGHV3-64*01	1,321	1,022	2,343	56.4	84	270	31.1	*P* < 10^−20^	*P* < 10^−20^
IGHV3-66*01	951	2,740	3,691	25.8	35	267	13.1	*P* < 10^−20^	*P* < 10^−20^
IGHV3-72*01	1,142	2,444	3,586	31.8	44	276	15.9	*P* < 10^−20^	*P* < 10^−20^
IGHV3-73*01	1,192	1,412	2,604	45.8	72	276	26.1	*P* < 10^−20^	*P* < 10^−20^
IGHV3-74*01	3,855	6,355	10,210	37.8	49	270	18.1	*P* < 10^−20^	*P* < 10^−20^
IGHV4-4*02	529	6,769	7,298	7.2	14	270	5.2	3.09 × 10^−14^	3.41 × 10^−14^
IGHV4-30-2*01	382	3,862	4,244	9.0	16	273	5.9	3.01 × 10^−16^	3.50 × 10^−16^
IGHV4-30-4*01	451	3,236	3,687	12.2	22	273	8.1	1.89 × 10^−18^	2.32 × 10^−18^
IGHV4-31*01	455	1,632	2,087	21.8	35	273	12.8	*P* < 10^−20^	*P* < 10^−20^
IGHV4-34*01	1,746	25,944	27,690	6.3	16	267	6.0	1.51 × 10^−2^	1.51 × 10^−2^
IGHV4-38-2*01	294	1,739	2,033	14.5	24	270	8.9	1.96 × 10^−16^	2.35 × 10^−16^
IGHV4-39*01	3,202	8,585	11,787	27.2	38	273	13.9	*P* < 10^−20^	*P* < 10^−20^
IGHV4-59*01	2,085	15,502	17,587	11.9	20	267	7.5	*P* < 10^−20^	*P* < 10^−20^
IGHV4-61*01	438	2,059	2,497	17.5	27	273	9.9	*P* < 10^−20^	*P* < 10^−20^
IGHV5-10-1*03	694	2,589	3,283	21.1	39	270	14.4	*P* < 10^−20^	*P* < 10^−20^
IGHV5-51*01	2,584	12,755	15,339	16.8	34	270	12.6	*P* < 10^−20^	*P* < 10^−20^
IGHV6-1*01	3,610	8,402	12,012	30.1	51	279	18.3	*P* < 10^−20^	*P* < 10^−20^
IGHV7-4-1*01	1,394	3,237	4,631	30.1	43	270	15.9	*P* < 10^−20^	*P* < 10^−20^

*Expected mutation probability (“Percent WGCW/WA Sites”) is (number of WCGW/WA sites)/(IGHV gene length ungapped). Observed fraction of mutations in (“Percent Mutations in WGCW/WA sites”) WGCW/WA sites is (number of mutations in WGCW/WA sites)/(total mutations). P-value is for binomial test, both raw and FDR-corrected (see Materials and Methods)*.

### Patterns of Co-localized Overlapping AID and Polη Hotspots Characterize Variation in CDRs 1 and 2 Across IGHV Genes

**Figure 4:**
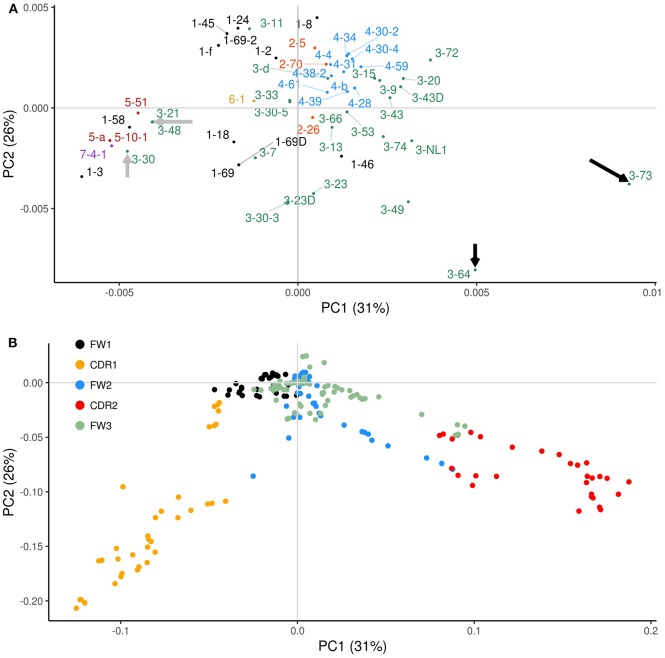
PCA analysis of overlapping AID (WGCW) and Polη (WA/TW) hotspots. Plots equivalent to Figure 2 but using the combined overlapping AID and Polη (WGCW/WA) hotspot distributions. **(A)** Corresponding PCA scores of the co-localized profiles. Gray arrows point to IGHV genes with co-localized profiles enriched in CDR1; and black arrows indicate IGHV genes with an especially strong co-localization signal focused in CDR2. **(B)** Corresponding PCA loadings colored according to relevant sub-region. Gene labels located far from their corresponding dot are attached by a fine line to compensate for overlapping nearby labels.

We repeated the PCA analysis but now examined the profiles of co-localized overlapping AID hotspots and Polη hotspots. Similarly to what we had observed previously using the AID WGCW distribution profiles (Figure 2A), IGHV genes tended to cluster by families after PCA transformation of the individual co-localization profiles (Figure 4A). In comparison to the PCA analysis using only WGCW sites (Figure 2B), here the PCA loadings revealed sites in CDR1 and CDR2 to be the foremost contributors to variation (Figure 4B). We also observed little contribution of FW1 sites to overall variation, due to Polη hotspots mostly being absent from the recurrent sub-region of AID overlapping hotspots in the 5′ end of FW1 (Figures S1, S2). FW3 sites similarly appear to have a reduced contribution to variation, however, the co-localization of WGCW and Polη sites that does occur appears to be due to the sites at around positions 250–275 in FW3 that also contain recurrent hotspots (Figures S1, S2).

Co-localization of AID WGCW and Polη hotspots therefore appears to focus on CDRs 1 and 2 as key drivers of variation between IGHV genes. Interestingly, the loadings (Figure 4B) for CDR1 and CDR2 appear to be orthogonal (extending out at approximately right angles to each other), which further suggests that the patterns between CDR1 and CDR2 are independent. To analyze this further, we used the co-localization profiles to calculate the mean values within CDR1 and CDR2, thus obtaining two values for each IGHV gene that represent the level of WGCW and WA co-localization for CDR1 and CDR2. We then calculated the correlation between the CDR1 and CDR2 values across all IGHV genes, and found it was not significant (Pearson's ρ = −0.09, *P* = 0.51; data not shown), consistent with the PCA loadings. Also consistent with these observations, although many IGHV genes have co-localized AID overlapping and Polη hotspots in both CDR1 and CDR2, several have the pattern solely in CDR1 or CDR2. The PCA scores highlight some of these cases (Figure 4A). Within the IGHV3 family, for example, IGHV3-21, IGHV3-48, and IGHV3-30 (gray arrows on the left side of the plot of Figure 4A) have the co-localization pattern in CDR1 only, and not in CDR2 (Figure S6A). In contrast, other IGHV3 family genes such as IGHV3-64 and 3-73 (black arrows on the extreme right side of Figure 4A) have co-localization profiles in both CDRs, but a strong signal predominantly in CDR2. IGHV3-73, in particular, has four overlapping hotspots, an exceptionally high number, in or adjoining CDR2 and in the 5′ region of FW2 together with many WA sites (Figure S6B).

### Non-functional IGHV Genes Have Reduced Overlapping Hotspots in the CDRs

**Figure 5:**
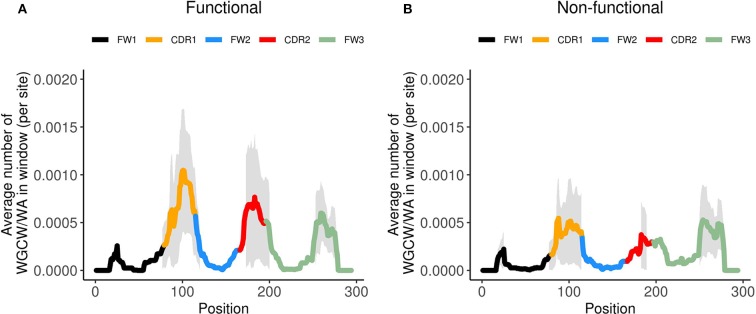
Co-localized WGCW/WA profiles for functional and non-functional IGHV genes. Site-by-site calculation of the average number of WGCW/WA co-localized hotspots of found in a window of size 31 (+/– 15 nt around each site) for **(A)** functional IGHV genes and **(B)** non-functional IGHV genes. The bold line indicates the average across the respective genes and is colored according to sub-region. The shaded region represents +/– 1 standard deviation at each site.

Thus far we only considered genes defined by IMGT as functional. However, the human IGHV locus contains many non-functional genes, which can be categorized as either pseudogenes or open reading frames (ORFs). Pseudogenes contain coding region stop codon(s) and/or frameshift mutations, whereas ORFs contain alterations in splicing sites, recombination signals and/or regulatory elements leading to non-functionality (33). To gain further insight into the possible evolutionary causes of loss of functionality in IGHV genes, we generated co-localization profiles for the 32 non-functional genes. The co-localization profiles of functional genes (Figure 5A) and non-functional genes (Figure 5B) revealed a striking difference in the magnitude of the WGCW/WA signal located in the CDRs, with functional genes having a signal about double the magnitude as non-functional genes in both CDR1 and 2. On the other hand, there appears to be no change in the co-localization signal in FW3 (Figure 5). As a consequence of the reduction, for non-functional genes, in the number of sub-regions containing AID overlapping and Polη hotspots in the CDRs (Figure S7), we can speculate that this then leads to an increased relative importance of FW3 (Figure 5B). Although non-functional genes will accumulate mutations as they evolve, due to the absence of selection we might assume they are similar to what they were in their last stages of functionality, i.e., before becoming non-functional. Under this assumption, our results would suggest that having co-localized AID overlapping and Polη hotspots in the CDRs is a contributor to evolutionary survival for an IGHV gene.

## Discussion

Since the discovery of AID (34), many details of the mechanisms associated with somatic hypermutation of antibody V regions have been elucidated, in particular, the roles of ncBER and ncMMR repair mechanisms that act downstream of the original AID-mediated mutation (3, 6, 7, 35). Biochemical characterization of AID and of Polη, a key polymerase involved in ncMMR, led to a better understanding of their preferences for the hotspots (WRC and WA respectively) (3). Further analyses of unselected *in vivo* data from the human IGHV3-23 gene confirmed that overlapping hotspots, defined by the hotspot WGCW, were particularly highly mutated in human IGHV genes (25). Analysis of the same IGHV3-23 dataset by ourselves showed that mutations in two particular WGCW hotspots were associated with mutations throughout the V gene, and that these highly mutated WGCW sites tended to be close to clusters of Polη (WA/TW) hotspots, a simple feature we suggested might contribute to higher mutability (24).

While the nucleotide differences in the 56 functional human IGHV genes, as well as the additional 32 non-functional genes, are obvious, the forces that have driven those differences are still unclear, and we do not understand how exactly they relate to each other and have co-evolved in terms of mutability. This is of particular importance because the special characteristics of the germline sequences that have evolved and been retained allow us to make antibodies to every possible foreign substance and infectious agent (36). To begin to address this question, we generated profiles describing the frequencies of AID overlapping hotspots (WGCW) for all functional human IGHV genes, and identified regions of high frequency not only in the CDRs, as would be expected, but also in a sub-region of FW1 and another in FW3 that has been referred to as “CDR4” (Figure 1B) (29). Beyond the characteristics of the CDRs and the FWs, there are clear sequence differences and patterns between the families and within each family that can be seen visually (Figures S1, S2). However, it has not been possible to determine which of these differences are most important in driving the evolution of the germline V region sequences to produce the antibody diversity that we require while retaining the structural integrity of the antibody structure. We therefore applied Principal Components Analysis (PCA), a form of dimensionality reduction that works to maximally capture the variance of the original data, to the WGCW profiles for all functional IGHV genes. We first found that, when comparing the distribution of AID overlapping hotspots, most variation in the hotspot distribution—as reflected in the loadings for the first two principal components (Figure 2B)—was primarily dependent on sites in FW3 (together with some sites in FW1) and in CDR1. The tight cluster of overlapping hotspots at the 5′ end of FW1 is particularly interesting because it is so highly conserved within each of the families except IGHV2 where it is not present (Figures S1, S2). This sub-region is at the very N-terminal end of the V region and, based on the crystal structure of IGHV3-23^*^01, is unlikely to come in direct contact with antigen (24). This sub-region is also rich in AGCTs and, again in IGHV3-23^*^01, these are mutated in many V genes but at a much lower frequency than the AGCTs in other parts of the gene (24). Furthermore, in IGHV3-23^*^01 sequences that have not undergone mutations at those sites, there is an overall lower frequency of somatic mutation throughout the gene compared to IGHV genes in which they have been mutated, suggesting that they do have some association with the AID mutational process (24). We also do not understand the role of the overlapping TGCA AID hotspots that are in FW1 just 5′ to CDR1 in all of the IGHV1, 2, and almost all IGHV3 family members, but missing from some IGHV5 and IGHV4 family members (Figures S1, S2). This site is not highly mutated in any of those families, but the fact that it is so highly retained and always a TGCA motif suggests it does have some role.

The number of functional IGHV genes varies greatly between the seven different IGHV families with IGHV6 and IGHV7 having only one productive germline gene each, and the IGHV3 family containing the most, with 25 functional members. The reasons for these differences are unclear. In our analysis of WGCW hotspots, variation in CDR2 appeared to be relatively low although, interestingly, the loadings along the first principal component (PC1) for FW3 (together with some sites in FW1) and CDR2 are located in opposing directions, indicating that their patterns of WGCW hotspots were negatively correlated. The major differences between IGHV families along PC1, in terms of overlapping hotspots, were found primarily in the FW3 (“CDR4”) and FW1 sites, whereas the loadings along PC2 largely corresponded to CDR1 sites. Since, by definition, PC1 and PC2 are independent, the pattern of WGCW sites in CDR1 was uncorrelated with that of CDR2, as well as FW1/FW3 (Figures S5C,D). It was also evident that CDR1 mainly contributed to the hotspot variation seen between IGHV genes that are members of the same family (intra-family differences). We can further speculate that the pattern of WGCW sites in the FW1/FW3 sub-region that is largely conserved within each family, may facilitate different levels of hotspot evolution within the family. Different families indeed appear to have different levels of variation. For example, if we consider the three largest IGHV families (see Figure S8A, showing variation for the IGHV1, 3 and 4 families), the IGHV1 and IGHV3 families tend toward greater variation in the CDRs than does IGHV4 (Figure S8B, shows corresponding variation averages for each FW and CDR sub-regions).

We next considered the potential for interactions between AID overlapping (WGCW) and Polη (WA/TW) hotspots. We defined a basic measure for co-localization of AID and Polη hotspots, and then used it to define profiles for the 56 functional human IGHV genes. Using high-throughput mutation data, we verified that sites contained within co-localized WGCW/WA sub-regions were indeed more significantly mutated in the majority of human IGHV genes. Analysis of the co-localization profiles revealed a pattern whereby CDR1 and CDR2 were the primary contributors to variation, as one might expect given the increased potential of the CDRs for interaction with antigen. In comparison, FW3 was less impactful and FW1 did not seem to play a role at all, which contrasted with the previous observations on the WGCW-only profiles. The relative importance of “CDR4” sub-region of FW3 in the WGCW-only profiles may be related to a previously proposed mutation model (37, 38) where co-occurring *pairs* of mutations demarcate sub-regions that are subsequently repaired by ncMMR. Under this model, WA hotspots *between* highly targeted AID hotspots (e.g., in CDR2) would presumably be targeted by Polη, while the original AID mutations at the boundaries (e.g., in FW1 or “CDR4”) might be repaired. Further mutations at AID hotspots may be possible within the exposed ssDNA patch due to the greater efficiency of UNG activity on ssDNA (39). The mutation pair model may also explain the results we observed when analyzing WGCW sites alone (Figure 2B), which shows CDR2 as less variable than “CDR4” and CDR1, since paired mutations in “CDR4” and CDR1 might lead to CDR2 mutations when the ssDNA patch includes CDR2. Interestingly, the three IGHV3 family genes, IGHV3-21, IGHV3-48, and IGHV3-30 highlighted above as having no overlapping hotspots in CDR2 (gray arrows on the left side of the plot of Figure 4A) do, however, have a high abundance of WA/TW sites in CDR1 (Figure S6A). At the same time, mutations in “CDR4” may be selected for directly, as appears to be the case for anti-influenza broadly-neutralizing antibodies, where FW3 residues form a loop that interacts directly with antigen (40, 41).

When we further incorporated non-functional genes (i.e., open reading frame genes and pseudogenes) into the analysis, the overall co-localized WGCW/WA signal was markedly reduced in both CDRs, while the variation in FW3 remained roughly the same in comparison to the functional genes. A characteristic observed in many of the non-functional genes is an absence of AID overlapping hotspots in their CDRs (Figure S7), which may explain their loss in functionality, and perhaps can give us further insight into predicting future pseudogenes from the pool of presently functional IGHV genes (for example, IGHV1-8, which lacks WGCW sites in either CDR1 or CDR2).

In summary, a simple measure describing the co-localization of WGCW and WA/TW hotspots appears to capture key aspects of variation of mutability in human IGHV genes, and suggests that this co-localization in highly mutating sub-regions such as the CDRs has contributed to shaping the evolution of the human IGHV genes.

## Data Availability Statement

The datasets generated for this study can be found in the NCBI SRA BioProject IDs 381394, 591804 (https://www.ncbi.nlm.nih.gov/bioproject/381394; http://www.ncbi.nlm.nih.gov/bioproject/591804).

## Author Contributions

CT and TM designed the research and methods. NC and DB provided the data. DB pre-processed the data. CT and TM performed the data analysis. CT, MS, and TM prepared the manuscript. All co-authors read and approved manuscript.

## Conflict of Interest

The authors declare that the research was conducted in the absence of any commercial or financial relationships that could be construed as a potential conflict of interest.
