# Supplementary material for: AID Overlapping and Polη Hotspots Are Key Features of Evolutionary Variation Within the Human Antibody Heavy Chain (IGHV) Genes
Source: Front Immunol. 2020 Apr 30;11:788. doi: 10.3389/fimmu.2020.00788 (PMC7204545; doi:10.3389/fimmu.2020.00788)
Supplement: Supplementary file 1 [file Data_Sheet_1.docx]

**Table S1.** Binomial test results showing mutability of WGCW/WA sites (CDRs). Results for CDR sub-regions follow same format as **Table 1**.

| ALLELE | NUM MUTATIONS WITHIN  WGCW/WA REGIONS | NUM MUTATIONS OUTSIDE  WGCW/WA REGIONS | TOTAL NUM MUTATIONS | PERCENT MUTATIONS  WITHIN  WGCW/WA REGIONS | NUM WGCW/WA  SITES | CDR LENGTH (UNGAPPED) | PERCENT WGCW/WA SITES | P-VALUE | CORRECTED P-VALUE |
| --- | --- | --- | --- | --- | --- | --- | --- | --- | --- |
| IGHV1-2*02 | 960 | 2741 | 3701 | 25.9 | 10 | 48 | 20.8 | 5.57 x 10^-14^ | 1.31 x 10^-13^ |
| IGHV1-3*01 | 1747 | 1392 | 3139 | 55.7 | 21 | 48 | 43.8 | P < 10^-20^ | P < 10^-20^ |
| IGHV1-8*01 | 422 | 2064 | 2486 | 17.0 | 7 | 48 | 14.6 | 5.07 x 10^-4^ | 6.99 x 10^-04^ |
| IGHV1-18*01 | 3913 | 2225 | 6138 | 63.8 | 31 | 48 | 64.6 | 9.16 x 10^-1^ | 9.90 x 10^-1^ |
| IGHV1-46*01 | 2615 | 697 | 3312 | 79.0 | 34 | 48 | 70.8 | P < 10^-20^ | P < 10^-20^ |
| IGHV1-58*01 | 331 | 302 | 633 | 52.3 | 24 | 48 | 50.0 | 1.33 x 10^-1^ | 1.66 x 10^-1^ |
| IGHV1-69*01 | 2327 | 414 | 2741 | 84.9 | 35 | 48 | 72.9 | P < 10^-20^ | P < 10^-20^ |
| IGHV2-5*01 | 130 | 977 | 1107 | 11.7 | 7 | 51 | 13.7 | 9.77 x 10^-1^ | 1.00 |
| IGHV2-26*01 | 857 | 239 | 1096 | 78.2 | 37 | 51 | 72.5 | 1.08 x 10^-5^ | 2.06 x 10^-5^ |
| IGHV3-7*01 | 3881 | 1827 | 5708 | 68.0 | 34 | 48 | 70.8 | 1.00 | 1.00 |
| IGHV3-9*01 | 3715 | 3175 | 6890 | 53.9 | 18 | 48 | 37.5 | P < 10^-20^ | P < 10^-20^ |
| IGHV3-13*01 | 2875 | 745 | 3620 | 79.4 | 29 | 45 | 64.4 | P < 10^-20^ | P < 10^-20^ |
| IGHV3-15*01 | 1153 | 1525 | 2678 | 43.1 | 19 | 54 | 35.2 | 2.48 x 10^-17^ | 7.08 x 10^-17^ |
| IGHV3-20*01 | 477 | 478 | 955 | 49.9 | 18 | 48 | 37.5 | 3.41 x 10^-15^ | 8.53 x 10^-15^ |
| IGHV3-21*01 | 2793 | 3675 | 6468 | 43.2 | 21 | 48 | 43.8 | 8.25 x 10^-1^ | 9.16 x 10^-1^ |
| IGHV3-23*01 | 15541 | 994 | 16535 | 94.0 | 45 | 48 | 93.8 | 1.05 x 10^-1^ | 1.35 x 10^-1^ |
| IGHV3-30-3*01 | 2363 | 397 | 2760 | 85.6 | 40 | 48 | 83.3 | 5.81 x 10^-4^ | 7.75 x 10^-4^ |
| IGHV3-30*01 | 367 | 554 | 921 | 39.8 | 19 | 48 | 39.6 | 4.47 x 10^-1^ | 5.11 x 10^-1^ |
| IGHV3-33*01 | 3247 | 3011 | 6258 | 51.9 | 21 | 48 | 43.8 | P < 10^-20^ | P < 10^-20^ |
| IGHV3-43*01 | 469 | 370 | 839 | 55.9 | 24 | 48 | 50.0 | 3.53 x 10^-4^ | 5.05 x 10^-4^ |
| IGHV3-48*01 | 876 | 1266 | 2142 | 40.9 | 21 | 48 | 43.8 | 9.96 x 10^-1^ | 1.00 |
| IGHV3-49*03 | 812 | 134 | 946 | 85.8 | 44 | 54 | 81.5 | 2.24 x 10^-4^ | 3.44 x 10^-4^ |
| IGHV3-53*01 | 1823 | 581 | 2404 | 75.8 | 29 | 45 | 64.4 | P < 10^-20^ | P < 10^-20^ |
| IGHV3-64*01 | 765 | 52 | 817 | 93.6 | 43 | 48 | 89.6 | 3.55 x 10^-5^ | 5.91 x 10^-5^ |
| IGHV3-66*01 | 801 | 223 | 1024 | 78.2 | 29 | 45 | 64.4 | P < 10^-20^ | P < 10^-20^ |
| IGHV3-72*01 | 831 | 412 | 1243 | 66.9 | 27 | 54 | 50.0 | P < 10^-20^ | P < 10^-20^ |
| IGHV3-73*01 | 648 | 170 | 818 | 79.2 | 36 | 54 | 66.7 | 1.56 x 10^-15^ | 4.15 x 10^-15^ |
| ALLELE | NUM MUTATIONS WITHIN  WGCW/WA REGIONS | NUM MUTATIONS OUTSIDE  WGCW/WA REGIONS | TOTAL NUM MUTATIONS | PERCENT MUTATIONS  WITHIN  WGCW/WA REGIONS | NUM WGCW/WA  SITES | CDR LENGTH (UNGAPPED) | PERCENT WGCW/WA SITES | P-VALUE | CORRECTED P-VALUE |
| IGHV3-74*01 | 2739 | 914 | 3653 | 75.0 | 32 | 48 | 66.7 | P < 10^-20^ | P < 10^-20^ |
| IGHV4-4*02 | 471 | 1721 | 2192 | 21.5 | 10 | 48 | 20.8 | 2.33 x 10^-1^ | 2.82 x 10^-1^ |
| IGHV4-30-2*01 | 350 | 877 | 1227 | 28.5 | 12 | 51 | 23.5 | 3.07 x 10^-5^ | 5.34 x 10^-5^ |
| IGHV4-30-4*01 | 422 | 601 | 1023 | 41.3 | 18 | 51 | 35.3 | 4.55 x 10^-5^ | 7.28 x 10^-5^ |
| IGHV4-31*01 | 346 | 221 | 567 | 61.0 | 25 | 51 | 49.0 | 6.23 x 10^-9^ | 1.25 x 10^-8^ |
| IGHV4-34*01 | 1476 | 5074 | 6550 | 22.5 | 10 | 45 | 22.2 | 2.76 x 10^-1^ | 3.25 x 10^-1^ |
| IGHV4-39*01 | 3036 | 962 | 3998 | 75.9 | 33 | 51 | 64.7 | P < 10^-20^ | P < 10^-20^ |
| IGHV4-59*01 | 1962 | 3157 | 5119 | 38.3 | 16 | 45 | 35.6 | 2.00 x 10^-5^ | 3.63 x 10^-5^ |
| IGHV4-61*01 | 425 | 335 | 760 | 55.9 | 23 | 51 | 45.1 | 1.41 x 10^-9^ | 2.97 x 10^-9^ |
| IGHV5-10-1*03 | 603 | 372 | 975 | 61.8 | 24 | 48 | 50.0 | 6.80^-14^ | 1.51 x 10^-13^ |
| IGHV5-51*01 | 2284 | 2437 | 4721 | 48.4 | 22 | 48 | 45.8 | 2.39 x 10^-4^ | 3.54 x 10^-4^ |
| IGHV6-1*01 | 1857 | 1407 | 3264 | 56.9 | 23 | 57 | 40.4 | P < 10^-20^ | P < 10^-20^ |
| IGHV7-4-1*01 | 943 | 446 | 1389 | 67.9 | 21 | 48 | 43.8 | P < 10^-20^ | P < 10^-20^ |

**Table S2.** Binomial test results showing mutability of WGCW/WA sites (FWs). Results for FW sub-regions follow same format as **Table 1**.

| ALLELE | NUM MUTATIONS WITHIN  WGCW/WA REGIONS | NUM MUTATIONS OUTSIDE  WGCW/WA REGIONS | TOTAL NUM MUTATIONS | PERCENT MUTATIONS  WITHIN  WGCW/WA REGIONS | NUM WGCW/WA  SITES | FW LENGTH (UNGAPPED) | PERCENT WGCW/WA SITES | P-VALUE | CORRECTED P-VALUE |
| --- | --- | --- | --- | --- | --- | --- | --- | --- | --- |
| IGHV1-3*01 | 633 | 8185 | 8818 | 7.2 | 10 | 246 | 4.1 | P < 10^-20^ | P < 10^-20^ |
| IGHV1-8*01 | 485 | 7497 | 7982 | 6.1 | 11 | 246 | 4.5 | 2.44 x 10^-11^ | 4.36 x 10^-11^ |
| IGHV1-18*01 | 3559 | 11722 | 15281 | 23.3 | 26 | 246 | 10.6 | P < 10^-20^ | P < 10^-20^ |
| IGHV1-24*01 | 76 | 3830 | 3906 | 2.0 | 3 | 246 | 1.2 | 8.30 x 10^-5^ | 1.26 x 10^-4^ |
| IGHV1-45*02 | 75 | 1844 | 1919 | 3.9 | 8 | 246 | 3.3 | 6.29 x 10^-2^ | 8.60 x 10^-2^ |
| IGHV1-46*01 | 1417 | 8627 | 10044 | 14.1 | 17 | 246 | 6.9 | P < 10^-20^ | P < 10^-20^ |
| IGHV1-58*01 | 128 | 2231 | 2359 | 5.4 | 9 | 246 | 3.7 | 1.10 x 10^-5^ | 1.74 x 10^-5^ |
| IGHV1-69*01 | 1072 | 6889 | 7961 | 13.5 | 19 | 246 | 7.7 | P < 10^-20^ | P < 10^-20^ |
| IGHV2-26*01 | 99 | 3447 | 3546 | 2.8 | 6 | 246 | 2.4 | 9.74 x 10^-2^ | 1.29 x 10^-1^ |
| IGHV3-7*01 | 850 | 13768 | 14618 | 5.8 | 6 | 246 | 2.4 | P < 10^-20^ | P < 10^-20^ |
| IGHV3-9*01 | 1477 | 16654 | 18131 | 8.2 | 12 | 246 | 4.9 | P < 10^-20^ | P < 10^-20^ |
| IGHV3-13*01 | 1474 | 11577 | 13051 | 11.3 | 19 | 246 | 7.7 | P < 10^-20^ | P < 10^-20^ |
| IGHV3-15*01 | 1393 | 6950 | 8343 | 16.7 | 26 | 246 | 10.6 | P < 10^-20^ | P < 10^-20^ |
| IGHV3-20*01 | 287 | 2417 | 2704 | 10.6 | 14 | 246 | 5.7 | P < 10^-20^ | P < 10^-20^ |
| IGHV3-21*01 | 653 | 12876 | 13529 | 4.8 | 7 | 246 | 2.9 | P < 10^-20^ | P < 10^-20^ |
| IGHV3-23*01 | 2313 | 37112 | 39425 | 5.9 | 9 | 246 | 3.7 | P < 10^-20^ | P < 10^-20^ |
| IGHV3-30-3*01 | 1544 | 5621 | 7165 | 21.5 | 28 | 246 | 11.4 | P < 10^-20^ | P < 10^-20^ |
| IGHV3-30*01 | 384 | 3231 | 3615 | 10.6 | 21 | 246 | 8.5 | 7.88 x 10^-6^ | 1.29 x 10^-5^ |
| IGHV3-33*01 | 3374 | 12710 | 16084 | 21.0 | 24 | 246 | 9.8 | P < 10^-20^ | P < 10^-20^ |
| IGHV3-43*01 | 439 | 1953 | 2392 | 18.4 | 28 | 246 | 11.4 | P < 10^-20^ | P < 10^-20^ |
| IGHV3-48*01 | 176 | 3472 | 3648 | 4.8 | 7 | 246 | 2.9 | 3.40 x 10^-11^ | 5.81 x 10^-11^ |
| IGHV3-49*03 | 620 | 2355 | 2975 | 20.8 | 38 | 246 | 15.4 | 3.46 x 10^-15^ | 7.09 x 10^-15^ |
| IGHV3-53*01 | 347 | 6472 | 6819 | 5.1 | 6 | 246 | 2.4 | P < 10^-20^ | P < 10^-20^ |
| IGHV3-64*01 | 556 | 1352 | 1908 | 29.1 | 41 | 246 | 16.7 | P < 10^-20^ | P < 10^-20^ |
| IGHV3-66*01 | 150 | 3151 | 3301 | 4.5 | 6 | 246 | 2.4 | 1.43 x 10^-12^ | 2.79 x 10^-12^ |
| IGHV3-72*01 | 311 | 2697 | 3008 | 10.3 | 17 | 246 | 6.9 | 2.28 x 10^-12^ | 4.25 x 10^-12^ |
| IGHV3-73*01 | 544 | 1766 | 2310 | 23.5 | 36 | 246 | 14.6 | P < 10^-20^ | P < 10^-20^ |
| IGHV3-74*01 | 1116 | 7316 | 8432 | 13.2 | 17 | 246 | 6.9 | P < 10^-20^ | P < 10^-20^ |
| IGHV4-4*02 | 58 | 6319 | 6377 | 0.9 | 4 | 246 | 1.6 | 1.00 | 1.00 |
| IGHV4-30-2*01 | 32 | 3797 | 3829 | 0.8 | 4 | 246 | 1.6 | 1.00 | 1.00 |
| IGHV4-30-4*01 | 29 | 3245 | 3274 | 0.9 | 4 | 246 | 1.6 | 1.00 | 1.00 |
| IGHV4-31*01 | 109 | 1735 | 1844 | 5.9 | 10 | 246 | 4.1 | 9.48 x 10^-5^ | 1.39 x 10^-4^ |
| IGHV4-34*01 | 270 | 26495 | 26765 | 1.0 | 6 | 246 | 2.4 | 1.00 | 1.00 |
| IGHV4-38-2*01 | 30 | 1625 | 1655 | 1.8 | 4 | 246 | 1.6 | 2.99 x 10^-1^ | 3.83 x 10^-1^ |
| IGHV4-39*01 | 166 | 9333 | 9499 | 1.8 | 5 | 246 | 2.0 | 9.79 x 10^-1^ | 1.00 |
| IGHV4-59*01 | 123 | 15085 | 15208 | 0.8 | 4 | 246 | 1.6 | 1.00 | 1.00 |
| IGHV4-61*01 | 13 | 2081 | 2094 | 0.6 | 4 | 246 | 1.6 | 1.00 | 1.00 |
| IGHV5-10-1*01 | 31 | 2060 | 2091 | 1.5 | 15 | 246 | 6.1 | 1.00 | 1.00 |
| IGHV5-51*01 | 300 | 12222 | 12522 | 2.4 | 12 | 246 | 4.9 | 1.00 | 1.00 |
| IGHV6-1*01 | 1753 | 9735 | 11488 | 15.3 | 28 | 246 | 11.4 | P < 10^-20^ | P < 10^-20^ |
| IGHV7-4-1*01 | 451 | 3825 | 4276 | 10.5 | 22 | 246 | 8.9 | 1.82 x 10^-4^ | 2.57 x 10^-4^ |
