# Supplementary material for: AID Overlapping and Polη Hotspots Are Key Features of Evolutionary Variation Within the Human Antibody Heavy Chain (IGHV) Genes
Source: Front Immunol. 2020 Apr 30;11:788. doi: 10.3389/fimmu.2020.00788 (PMC7204545; doi:10.3389/fimmu.2020.00788)
Supplement: Supplementary file 2 [file Presentation_1.pptx]

## Slide 1
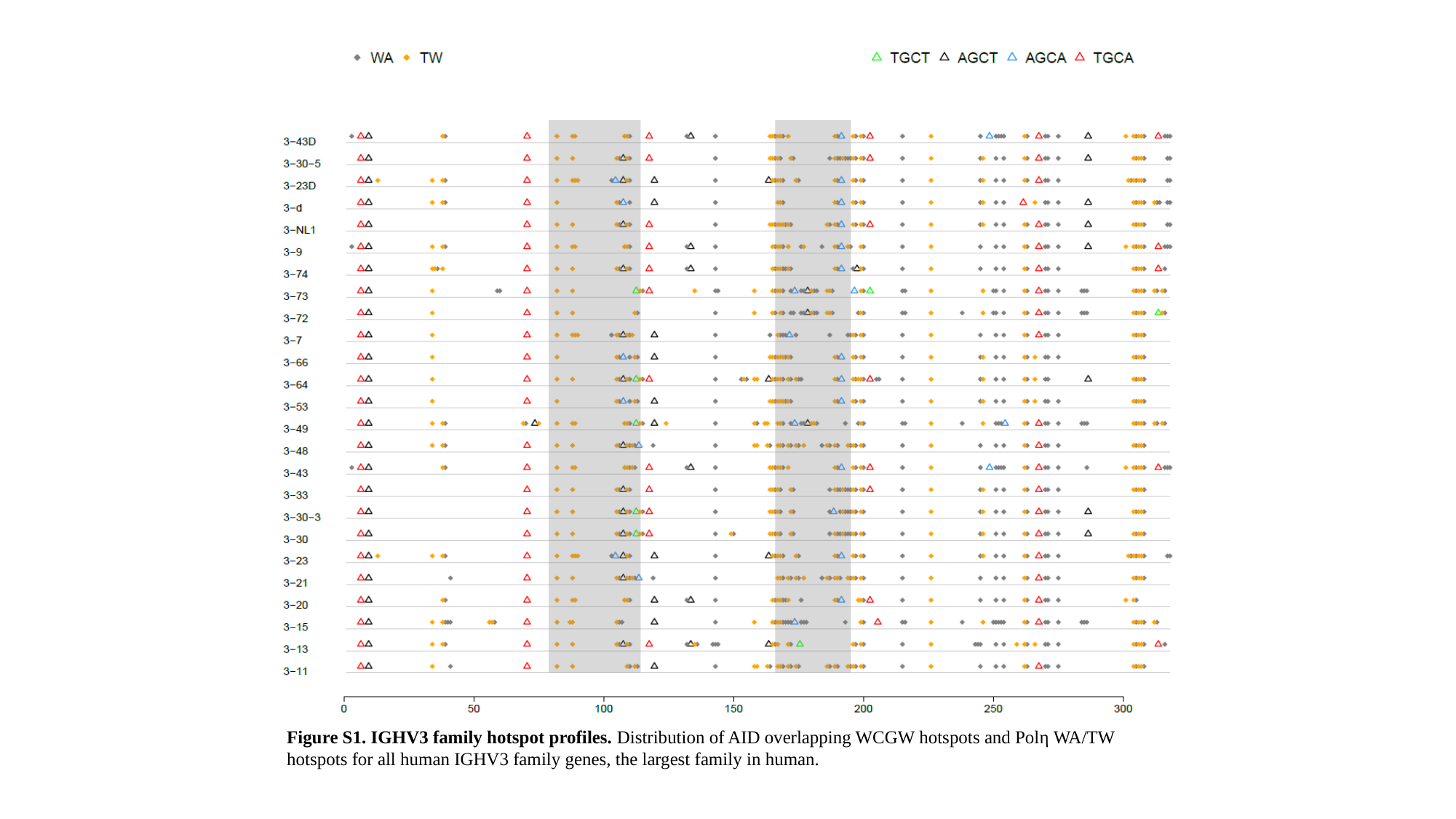

Figure S1. IGHV3 family hotspot profiles. Distribution of AID overlapping WCGW hotspots and Polη WA/TW hotspots for all human IGHV3 family genes, the largest family in human.

## Slide 2
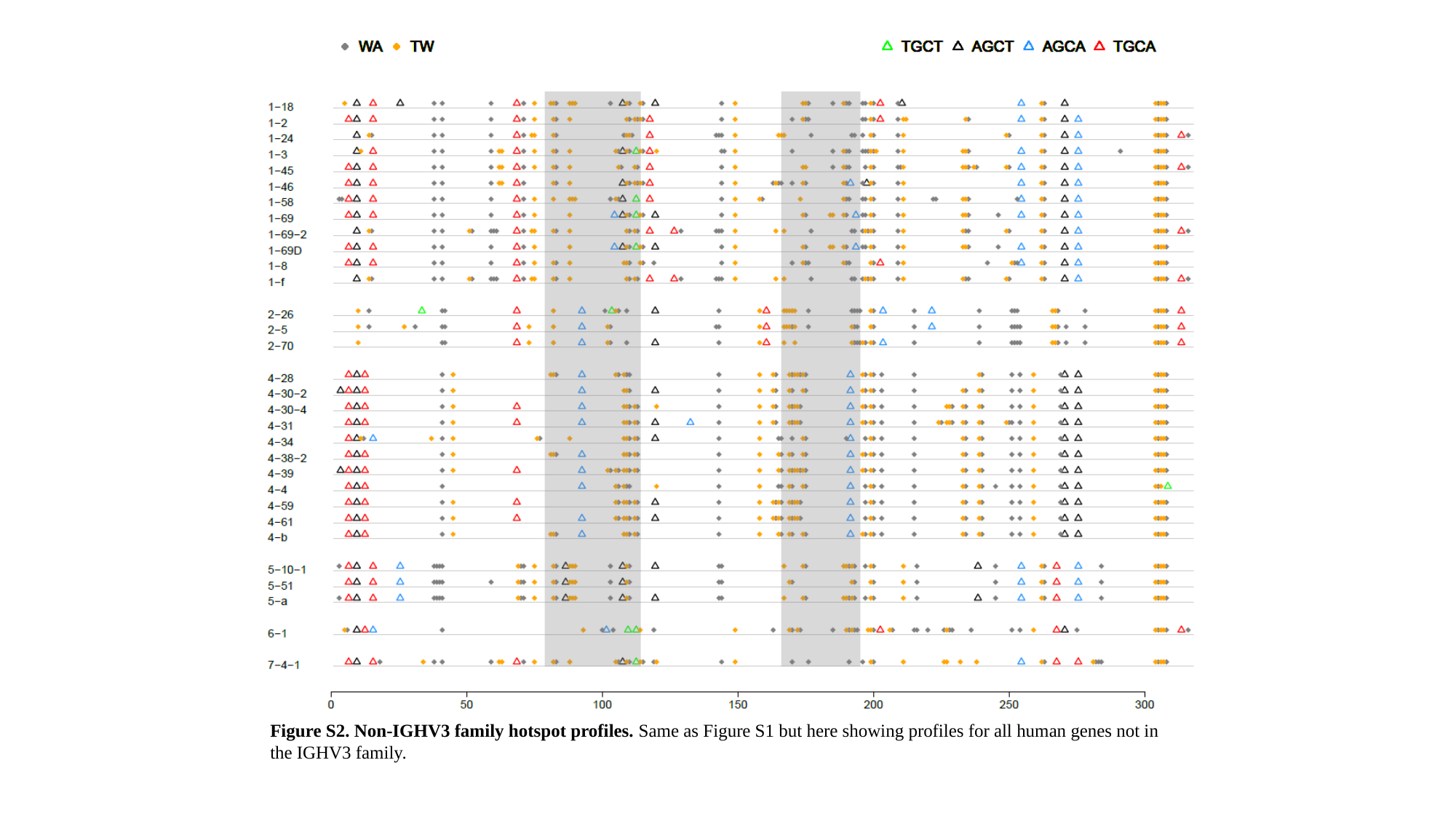

Figure S2. Non-IGHV3 family hotspot profiles. Same as Figure S1 but here showing profiles for all human genes not in the IGHV3 family.

## Slide 3
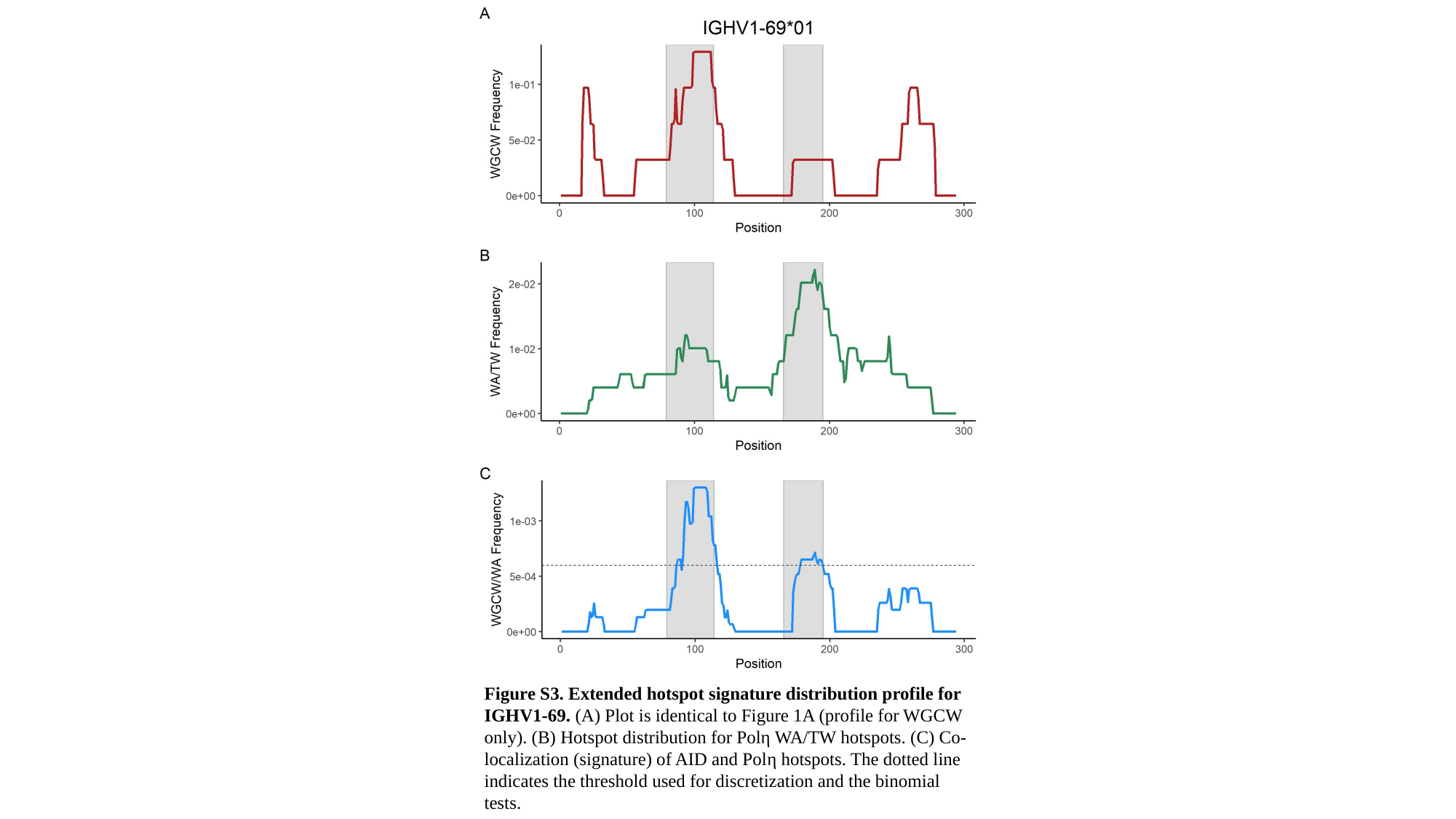

Figure S3. Extended hotspot signature distribution profile for IGHV1-69. (A) Plot is identical to Figure 1A (profile for WGCW only). (B) Hotspot distribution for Polη WA/TW hotspots. (C) Co-localization (signature) of AID and Polη hotspots. The dotted line indicates the threshold used for discretization and the binomial tests.

## Slide 4
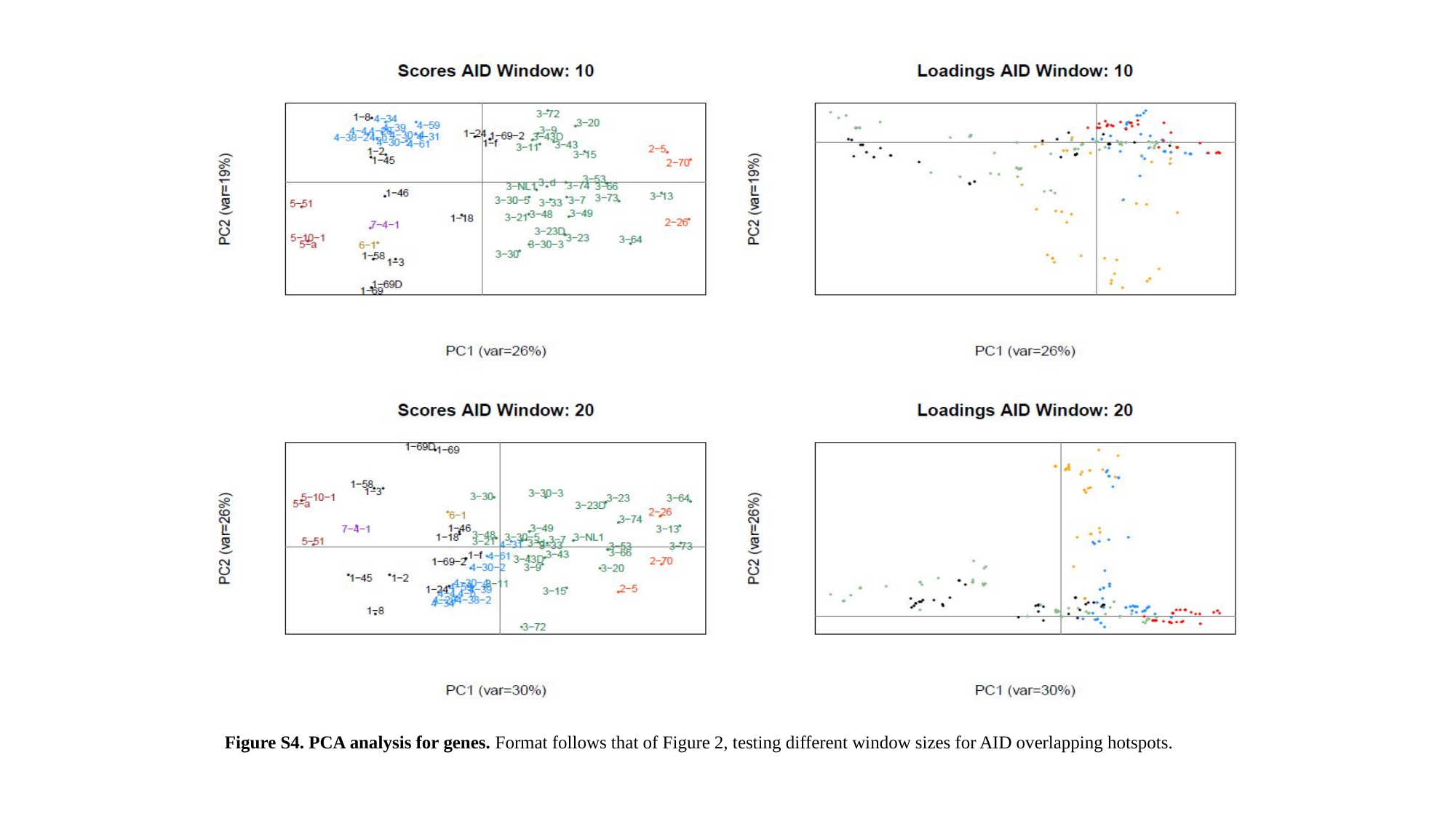

Figure S4. PCA analysis for genes. Format follows that of Figure 2, testing different window sizes for AID overlapping hotspots.

## Slide 5
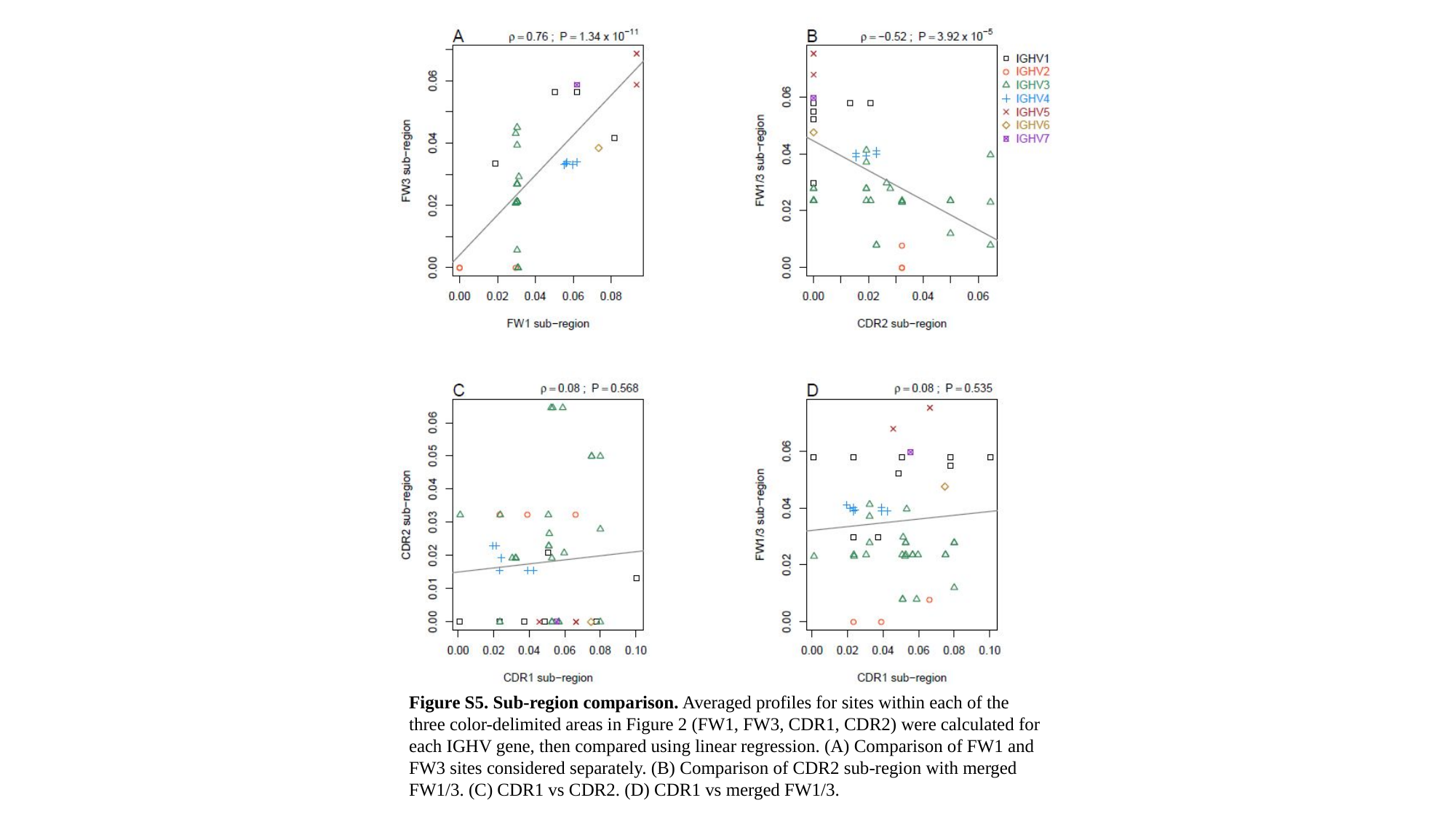

Figure S5. Sub-region comparison. Averaged profiles for sites within each of the three color-delimited areas in Figure 2 (FW1, FW3, CDR1, CDR2) were calculated for each IGHV gene, then compared using linear regression. (A) Comparison of FW1 and FW3 sites considered separately. (B) Comparison of CDR2 sub-region with merged FW1/3. (C) CDR1 vs CDR2. (D) CDR1 vs merged FW1/3.

## Slide 6
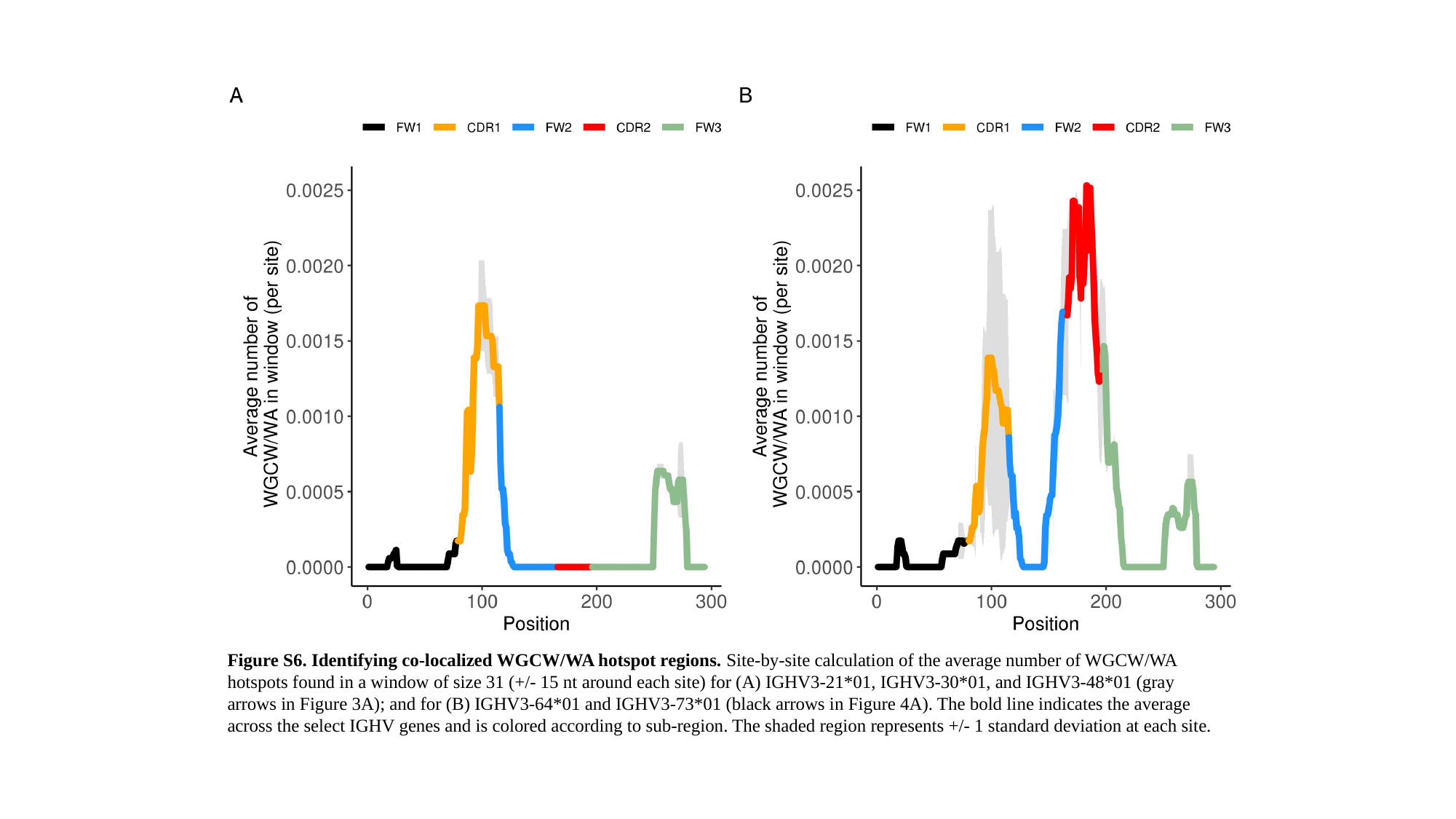

Figure S6. Identifying co-localized WGCW/WA hotspot regions. Site-by-site calculation of the average number of WGCW/WA hotspots found in a window of size 31 (+/- 15 nt around each site) for (A) IGHV3-21*01, IGHV3-30*01, and IGHV3-48*01 (gray arrows in Figure 3A); and for (B) IGHV3-64*01 and IGHV3-73*01 (black arrows in Figure 4A). The bold line indicates the average across the select IGHV genes and is colored according to sub-region. The shaded region represents +/- 1 standard deviation at each site.

## Slide 7
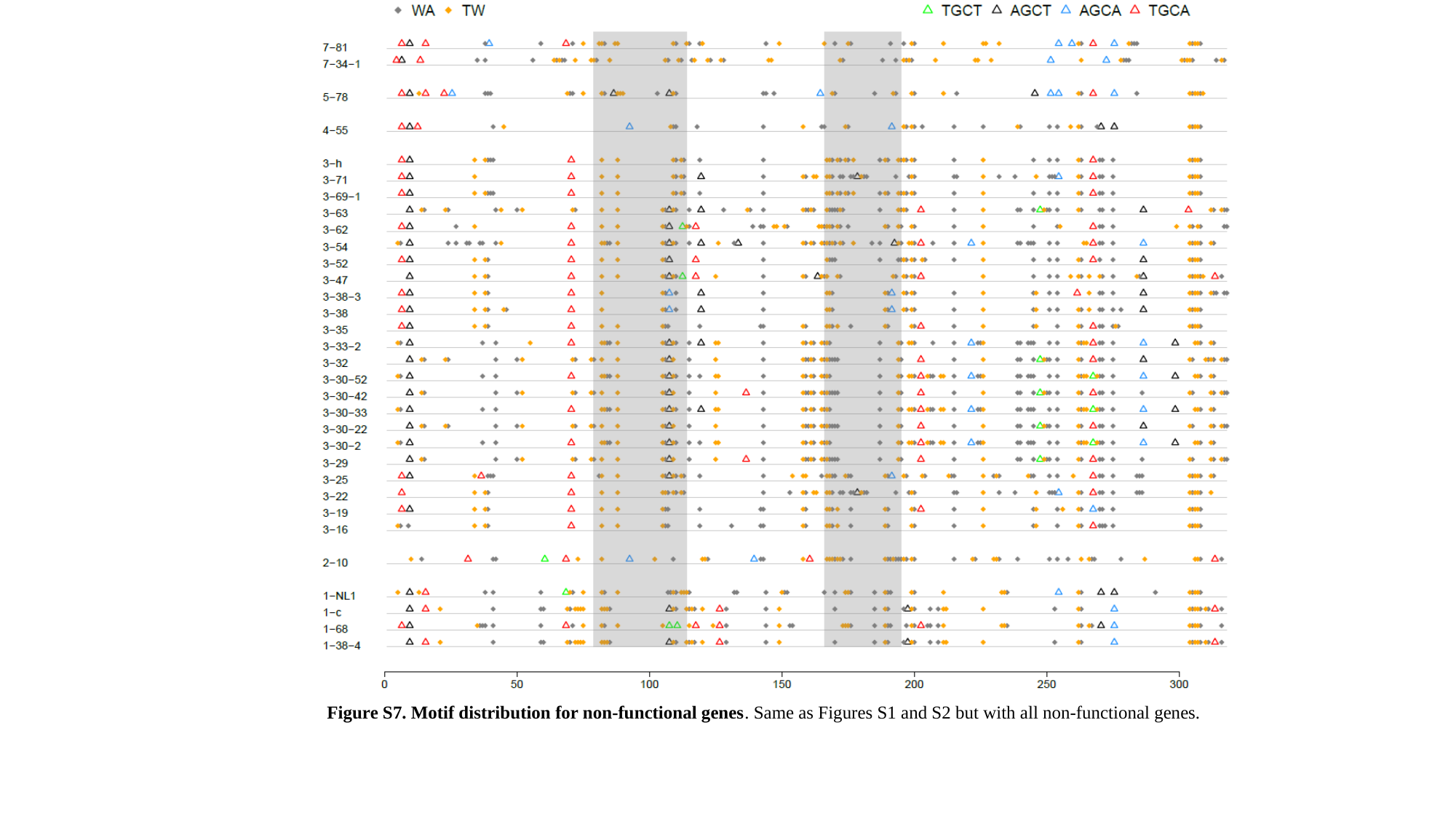

Figure S7. Motif distribution for non-functional genes. Same as Figures S1 and S2 but with all non-functional genes.

## Slide 8
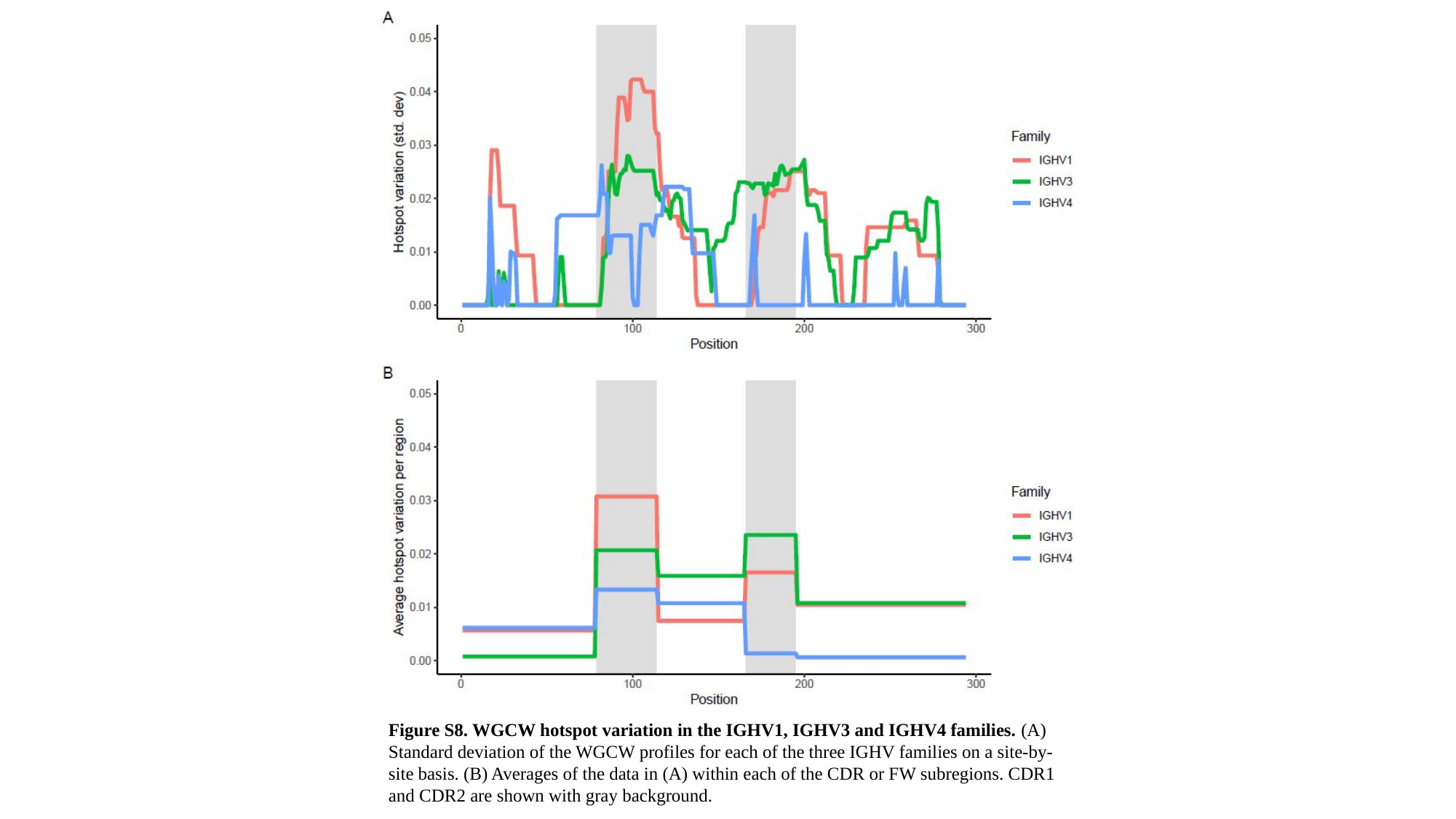

Figure S8. WGCW hotspot variation in the IGHV1, IGHV3 and IGHV4 families. (A) Standard deviation of the WGCW profiles for each of the three IGHV families on a site-by-site basis. (B) Averages of the data in (A) within each of the CDR or FW subregions. CDR1 and CDR2 are shown with gray background.
